# Effects of Volatile Components and Ethanolic Extract from *Eclipta prostrata* on Proliferation and Differentiation of Primary Osteoblasts

**DOI:** 10.3390/molecules15010241

**Published:** 2010-01-08

**Authors:** Xiong-Hao Lin, Yan-Bin Wu, Shan Lin, Jian-Wei Zeng, Pei-Yuan Zeng, Jin-Zhong Wu

**Affiliations:** 1 Academy of Integrative Medicine, Fujian University of Traditional Chinese Medicine, Fuzhou, Fujian 350108, China; E-Mails: linxionghao22@163.com (X.-H.L.); wxsq1@163.com (Y.-B.W.); 2 Department of Pharmacy, Fujian University of Traditional Chinese Medicine, Fuzhou, Fujian 350108, China

**Keywords:** *Eclipta prostrata*, volatile components, ethanolic extract, primary osteoblasts

## Abstract

*Eclipta prostrata*, an aromatic plant, is known in Chinese herbal medicine for the treatment of various kidney diseases. In the present study, the volatile components were isolated from the aerial parts of this plant by hydrodistillation and analysed by GC–MS. A total of 55 compounds, which were the major part (91.7%) of the volatiles, were identified by matching mass spectra with a mass spectrum library (NIST 05.L). The main components were as follows: heptadecane (14.78%), 6,10,14-trimethyl-2-pentadecanone (12.80%), *n*-hexadecanoic acid (8.98%), pentadecane (8.68%), eudesma-4(14),11-diene (5.86%), phytol (3.77%), octadec-9-enoic acid (3.35%), 1,2-benzenedicarboxylic acid diisooctyl ester (2.74%), (*Z,Z*)-9,12-octadecadienoic acid (2.36%), (*Z*)-7,11-dimethyl-3-methylene-1,6,10-dodecatriene (2.08%) and (*Z,Z,Z*)-1,5,9,9-tetramethyl-1,4,7-cycloundecatriene (2.07%). The effects of volatile components and ethanolic extract from the aerial parts of this plant on the proliferation and differentiation of primary osteoblasts were evaluated by the MTT method and measuring the activity of alkaline phosphatase (ALP activity). Both volatile components and ethanolic extract (1 μg/mL to 100 μg/mL) significantly (*p* < 0.01) stimulated the proliferation and increased the ALP activity of primary osteoblasts. These results propose that *E. prostrata* can play an important role in osteoblastic bone formation, and may possibly lead to the development of bone-forming drugs.

## Introduction

Bone formation and resorption is a balanced and continuous process. Osteoporosis, a disease characterized by low bone mass and microarchictectural deterioration of bone tissues, is due to the excess of osteoclastic bone resorption over osteoblastic bone formation [1,2,3]. Therefore, both stimulators of bone formation and specific suppressors on bone resorption are of therapeutic significance in the treatment of osteoporosis.

Recently, many antiosteoporotic agents such as estrogens, bisphosphonates, calcitonin, sodium fluoride and anabolic steroids have been developed to treat osteoporosis [4,5]. However, many of these agents do not produce bone mass of the desired quality or have undesirable side effects such as hypercalcemia, hypercalciurea, increased risk of endometrial and breast cancer, breast tenderness, menstruation, thromboembolic events, vaginal bleeding and hot flushes, which limit their clinical applications [6,7,8]. In addition, the most frequently used antiosteoporotic agents were developed in affluent countries and the costs are too high to benefit a large population in the developing or even developed countries for the prevention and treatment of osteoporosis [9]. Thus, it is indispensable to search for an effective, safe, and affordable antiosteoporotic drug. 

For more than a millennium, herbal medicine has been extensively used, apparently safely and effectively, in Asian countries, especially in China, Japan and Korea, to alleviate various symptoms of diseases [10,11,12,13,14]. So it will undoubtedly be a cost–effective alternative to commercial pharmaceutical products. The theory of traditional Chinese medicine believes that the normal physiological functions of the human body result from the opposite and unified relationship between Yin and Yang. Both of them are always in a state of dynamic balance. If, for any reason, the relative balance is destroyed, there is bound to be excess or deficiency of Yin or Yang, and then a disease will arise [15]. The theory of traditional Chinese medicine also believes that bones are governed and dominated by the “kidney”, which means that the “kidney” plays an important role in growth and formation of bones. Strong “kidney” can nourish bone and makes it flourish, but the weak “kidney” makes bone perish [16]. That is to say, both the deficiency of kidney Yin and the deficiency of kidney Yang can cause osteoporosis. *Eclipta prostrata* L. [syn: *Eclipta alba* (L) Hassk. Family: Astraceae], one of the oldest tonic herbs, is widely applied for its actions of tonifying the liver and kidney Yin, nourishing body’s essential fluid, and arresting hemorrhage. According to the above-mentioned theory, we hypothesized that *E. prostrata* might be able to prevent and treat osteoporosis for its efficacy of nourishing the kidney Yin. 

Various biological activities of the volatile components obtained from many plants have been reported [17,18,19]. So we believe that both volatile compoents and non-volatile components in herbs are responsible for their pharmacological activities [20,21]. *E. prostrata*, an aromatic plant, from which we could always smell the strong fragrance. Previous papers reported that the essential oils from the leaves and stem bark of this plant were analyzed respectively. All of 41 volatile compounds were detected [22]. The main components of the essential oil from the aerial parts of this plant were also investigated [23]. These results displayed significant difference on the qualitive and quantitative variations of essential oil. These differences were probably due to the different growth habitat [19]. As far as our literature survey could ascertain, there is no report on any pharmacological investigation on the volatile compoents of *E. prostrata*.

According to the above-mentioned theory and researches, in the present study, we identified the composition of the volatile components from the aerial parts of *E. prostrata* and investigated the effects of volatile components and ethanolic extract from the aerial parts of this plant on the proliferation and differentiation of primary osteoblasts. The results suggest that both volatile components and ethanolic extract could evidently (*p* < 0.01) facilitate proliferation and differentiation of primary cultural osteoblasts.

## Results and Discussion

Many Chinese tonic herbs have long been used for safeguarding health and delaying the onset of senility. According to traditional Chinese medicine theory, “kidney” controls bone. The “kidney-tonifying” action of traditional Chinese medicine might have relationship with bone formation [12,15]. *E. prostrata*, a typical “kidney-tonifying” traditional Chinese medicine, is widely used to prevent and treat various kidney diseases for its actions of nourishing the kidney Yin. In addition, pathological research has indicated that osteoporosis is associated with many factors. Oxidative stress has an important impact on osteoblast differentiation and mineralization [24]. Previous studies showed that the ethanol and ethyl acetate extracts of the leaves of *E. prostrata* displayed significantly antioxidative activity [25]. Therefore, we postulated that *E. prostrata* might be able to prevent and treat osteoporosis.

Osteoblastic bone formation is thought to be mediated by two different processes: one is the formation of new osteoblasts, and the other is the activity of osteoblasts to produce bone matrix. Osteoblast differentiation is a crucial aspect of bone formation and remodeling, a process that is severely compromised in osteoporosis. In bone tissues, the expression of alkaline phosphatase (ALP) is closely associated with osteoblastic differentiation and ALP is the most widely recognized biochemical marker for osteoblastic activity [13,26]. Although its precise mechanism of action is poorly understood, this enzyme is believed to play a role in bone mineralization [27]. 

*E. prostrata*, an aromatic plant, contains rich volatile components, which might be responsible for the pharmacological properties in part. Moreover, the quality and quantity of volatile components, related with pharmacological activities, are highly influenced by genetic and environmental factors [19]. So, in the present study, we identified the composition of the volatile components of *E.prostrata* and investigated the effects of volatile components and ethanolic extract from the aerial parts of this plant on the proliferation and ALP activity of primary osteoblasts.

### Volatile components analysis

All of 55 volatile compounds were identified. GC–MS profile of the volatile components showed the presence of a wide range of compounds, including terpenoids, aromatics, long-chain hydrocarbons, alcohols, aldehydes, ketones, acids and esters. The flavour compounds were constituted by non-terpenoids and terpenoids. Heptadecane (14.78%) was the main component of the oil, followed by 6,10,14-trimethyl-2-pentadecanone (12.80%), *n*-hexadecanoic acid (8.98%), pentadecane (8.68%), octadec-9-enoic acid (3.35%), 1,2-benzenedicarboxylic acid diisooctyl ester (2.74%) and (Z,Z)-9,12-octadecadienoic acid (2.36%). Among the terpenoids, eudesma-4(14),11-diene was present in a relatively high amounts (5.86%). Phytol (3.77%), (*Z*)-7,11-dimethyl-3-methylene-1,6,10-dodecatriene (2.08%) and (*Z,Z,Z*)-1,5,9,9-tetramethyl-1,4,7-cycloundecatriene (2.07%) were also present in comparable amounts.

The results revealed notable differences between our data and those previously reported for *E. prostrasta* [22]. The main components of both the essential oils from the leaves and stem bark of *E. prostrasta* were β-caryophyllene (47.7% and 15.9%) and α-humulene (31.8% and 12.9%). In addition, (*E*)-β-farnesene (10.0%) was also identified in significant amounts in the stem bark. These compounds were absent or only had low amount in the present study. Compared with the reported oils from the aerial parts of *E. prostrasta* [23], the main components in the oils in this study didn’t display significant differences. However, content differences of the main compoents were found. Moreover, some representative components of this plant such as 2,2':5',2''-terthiophene were detected in this study. Hence, we thought that the differences of volatile components might arise from several environmental (climatical, seasonal, and geographical) and genetic differences, which were the important factors influencing the quality of medicinal herbs.

**Figure 1:**
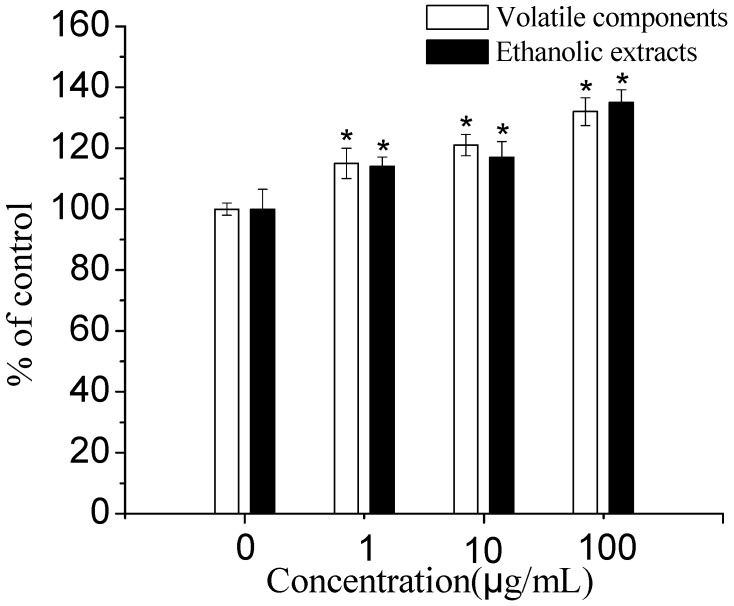
Effects of volatile components and ethanolic extract from *E. prostrata* on the proliferation of primary osteoblasts (n = 8, x ± SD; * *p* < 0.01, compared with control).

**Figure 2:**
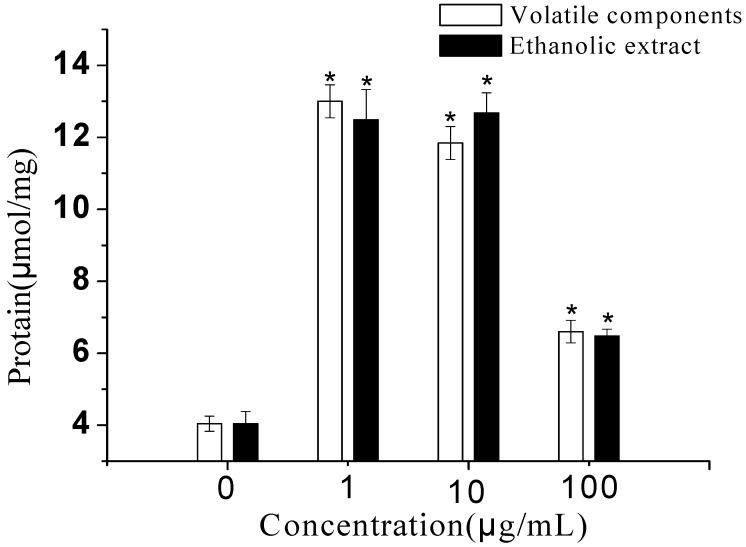
Effects of volatile components and ethanolic extract from *E. prostrata* on the ALP activity of primary osteoblasts (n = 8, x ± SD; * *p* < 0.01, compared with control).

### Proliferation and alkaline phosphatase activity assays

As the tested samples with different concentrations were added to wells for 48 h, both of which (1 μg/mL to 100 μg/mL) dose-dependently (*p* < 0.01) stimulated the proliferation of rat primary osteoblasts (Figure 1). To ascertain whether *E. prostrata* is capable of affecting osteoblastic cell differentiation, we examined the changes in ALP activity. As shown in Figure 2, both volatile components and ethanolic extract significantly (*p* < 0.01) increased ALP activity in osteoblasts over the 9 days, and the maximal effects of them were observed when cells were incubated with 1 μg/mL and 10 μg/mL, respectively. Therefore, *E. prostrata* could stimulate osteoblastic activity at least in part by enhancing synthesis of ALP.

Consistent with our study, some flavonoids isolated from the methanolic extract of *E. prostrata* have been reported to have antiosteoporotic activity. They significantly increased the ALP activity at concentrations ranging from 1.0 to 25.0 μM. However, these compounds did not show significant effects on osteoblast proliferation [28]. These results indicated that the ethanolic extract of *E. prostrata* might contain other antiosteoporotic ingredients, which could faciliate osteoblast proliferation. 

## Experimental

### Plant material

The aerial parts of *Eclipta prostrasta* L. [syn: *Eclipta alba* (L) Hassk. Family: Astraceae] (20080710) was purchased from Fujian Tianren Pharmaceutical Company and identified by Professor Cheng-zi Yang of the Department of Pharmacy, Fujian University of Traditional Chinese Medicine. The voucher specimens of these plants were deposited at the Herbarium of Department of Pharmacognosy, Fujian University of Traditional Chinese Medicine, Fuzhou, P.R. China.

### Chemicals and reagents

3-(4,5-Dimethylthiazol-2-yl)-2,5-diphenyltetrazolium bromide (MTT) and dimethyl suphoxide (DMSO) were purchased from Sigma (U.S.A.). Phenol red-free Dulbecco’s modified Eagle’medium (phenol red-free DMEM) and Fetal bovine serum (FBS) were purchased from Hyclone (U.S.A.). Ethanol, diethyl ether, anhydrous sodium sulphate, diethanolamine, disodium-4-nitrophenylphosphate, and 4-nitrophenol were of domestic AR grade.

### Extraction of volatile components

The material (200 g) was crushed (40 mesh), then soaked in 2,000 mL water for about 12 h before it was subjected to hydrodistillation in a Clevenger type apparatus. The contents were distilled for 3 h to obtain the volatile oil in a 0.24% (w/w) yield (on a dry mass) of yellowish colour and with a pleasant smell. The oils were dried over anhydrous sodium sulphate and stored at 4 °C in the dark until analyzed and tested.

### GC–MS analysis

GC–MS analysis was performed on an Agilent 6890N Network GC System, fitted with a HP-5MS capillary column (30 m × 0.25 mm i.d. × 0.25 μm film thickness; maximum temperature, 350 ^o^C), coupled to an Agilent 5975 inert XL Mass Selective Detector. Ultrahigh purity helium (99.999%) was used as carrier gas at a constant flow of 1.0 mL/min. The injection, transfer line and ion source temperatures were 250, 250 and 200 ^o^C, respectively. The ionizing energy was 70 eV. Electron multiplier (EM) voltage was obtained from autotune. All data were obtained by collecting the full-scan mass spectra within the scan range 35–500 amu. The splitless injection was employed for the analysis. The diluted sample (10 mg/mL, in redistilled diethyl ether) volume injected with an Agilent 7683B series injector was 1 μL. The oven temperature program was 90 °C–2.5 °C/min–130 °C–1.2 °C/min–170 °C–2 °C/min–230 °C–2 °C / min–250 °C (5 min).

### Identification and quantification of volatile components

Volatile components were first identified by comparing the spectra obtained with a mass spectrum library (NIST 05. L). Corroboration of the identification was then sought by matching the mass spectra of compounds with those in the literature and the retention times of the compounds reported on an equivalent column [19,22,23]. Component relative percentages were calculated from the TIC from the automated integrator. 

### Ethanolic extract

The powder of *E. prostrasta* (10 g) mixed with 75% (v/v) aqueous ethanol (100 mL) was loaded into a flask equipped with a water condenser tube. The extraction solvent was boiled (80 ± 2 °C) and refluxed for a period of 120 min. Extraction was repeated twice. The combined extracts were filtered through filter paper and evaporated to dryness in a rotary evaporator (RE-52, Shanghai Splendor and Biochemical Instrument Co., China) at 45 °C under reduced pressure to yield the crude ethanolic extract (1.3 g). 

### Preparation of test samples

Both volatile components and ethanolic extract were dissolved in dimethylsulfoxide (DMSO) at concentration of 10 mg/mL, and diluted in culture medium to the working solution before use. To avoid DMSO toxicity, the concentration of the solvent was less than 1% (v/v). For effects of steroids on growth or differentiation, culture media were charcoal stripped and without phenol red.

### Cell cultures

Sprague–Dawley rats, which were 2–3 days old, were purchased from the Experimental Animal Center of the Fujian Medical University, Fuzhou, P.R. China. Primary osteoblastic cells were prepared from the calvarias of newborn rats following the sequential enzymatic digestion method [29]. Briefly, skull (frontal and parietal bones) were dissected; then the endosteum and periosteum were stripped off, and the bone was cut into approximately 1–2 mm^2^ pieces and digested sequentially using trypsin (0.25%, w/v) for 30min and collagenase II (1.0 mg/mL) containing 0.05% trypsin (w/v) for 2 h. The cells were collected and cultured in phenol red free DMEM supplemented with 10% FBS and 1% penicillin/streptomycin, for 24 h in a humidified atmosphere of 5% CO_2_ in air at 37 °C, then media was changed.

### Assay for osteoblast proliferation and alkaline phosphatase (ALP) activity

The primary osteoblasts (2 × 10^4^ cells/well) were subcultured into 96-well culture plates, and incubated 24 h before the addition of test samples or control (DMSO, final concentration 1% v/v), then cultured for another 48 h. Prior to the end of culture, MTT (20 μL, 5 mg/mL) was added to each well and incubated for 4 h, after which the medium was discarded, and 150 µL of DMSO was added to each well. The cells were incubated for 20 min. The UV absorbance was measured at 490 nm at a microplate spectrophotometer (Bio-Rad Model 680, USA) at 490 nm with a reference at 630nm and used as an indicator of osteoblast proliferation. Proliferation (%) was calculated as 100 × (OD of volatile components or ethanolic extract-treated / OD of control), where OD is the average absorbance of six experiments with 8 replicates. Primary osteoblasts were seeded at 2 × 10^4^ cells/well in 96-well culture plates, and treated with test samples or control for 9 days (media were changed per three days). The ALP activity was measured according to the literature [30]. Total protein was assayed by the method of Bradford [31]. The activity of alkaline phosphatase was expressed as micromoles of 4-nitrophenol liberated per milligram protein.

### Statistical analysis

Data were expressed as the mean ± standard deviation. Statistical significances were analyzed by using the Student’s t-test. A value of *p* < 0.01 was considered significant. Linear regression analysis was performed by the correlation coefficient. 

## Conclusions

In the present study we have identified the volatile components from *E. prostrata* and evaluated the effects of volatile components and ethanolic extract from the aerial parts of this plant on the proliferation and ALP activity of primary osteoblasts. Our results showed that both volatile components and ethanolic extract could signiﬁcantly (*p* < 0.01) stimulate osteoblast proliferation in a dose-dependent manner and increased the ALP activity. Thus, it will be of interest to test further whether *E. prostrata* exert antiosteoporotic effects *in vivo*, for example, in animal models of osteoporosis, to explore its therapeutic potential. This will provide further insight into the design of new approaches to osteoporosis.
